# Consideration of Major Accidents and Disasters in Environmental Impact Assessment Reports for Natural Gas Pipeline Projects in Poland

**DOI:** 10.1007/s00267-025-02307-x

**Published:** 2025-12-02

**Authors:** Maria Teresa Markiewicz

**Affiliations:** Faculty of Environmental Engineering, Warsaw University of Technology, Warsaw, Poland

**Keywords:** Natural Gas Pipeline, Accidents and disasters, EIA, Risk assessment, Public participation, Social impacts

## Abstract

The analysis of the Polish environmental impact assessment (EIA) reports (EIARs) for natural gas (NG) projects, preceded by a legislation survey, with regards to the consideration of major accidents and disasters (MADs), showed that although reports prepared after January 1, 2017 contain more detailed information on MADs, none of the reports introduced the concept of risk, understood as the probability of hazards//impacts in combination with their consequences//effects, nor did they present the assessment results of such defined risk. The quality of the EIARs can be improved. It would be valuable, in addition to the arbitrary, minimum distances from the NG pipeline to other structures used in Poland (DMINs), to include assessment results of such defined risk. It is therefore recommended that sector-specific guidelines for assessing the risk of MADs on pipelines be developed in Poland to support LUP decisions, as well as general guidelines for integrating the risk assessment of MADs within the EIA. Although Polish EIA practitioners and proponents recognize the importance of public participation and consideration of social factors in the EIA practice, in the context of mitigating social conflicts fuelled by concerns about hazards posed by MADs, there is still room for improvement in this regard. It relates directly to the recommendations concerning the presentation of the results of the risk assessment of MADs in the EIARs. In addition, it is suggested that the Polish EIA legislation be modified and a set of man-made disasters analyzed in the EIA be expanded to include consideration of “technical disasters” in place of “construction disasters”.

## Introduction

Pipeline transport is a highly efficient and safe way of conveying NG (Natural Gas) from one location to another. Still, a pipeline failure can happen. Statistical data have shown an overall average failure rate in Europe of 0.277 per 1000 km from 1970 to 2022 and an average annual failure rate over the past five years of 0.101 per 1000 km (EGIG (European Gas Pipeline Incident Data Group) 2023). Accidental releases from pipelines can result from a variety of causes, including external (third-party) interference- which can be classified as unintentional (such as human error, excavation works) and intentional (such as sabotage) – and external corrosion, mechanical (structural and material) failure, internal corrosion and natural hazards, which according to Girgin and Krausmann (2014) can be categorized into meteorological, hydrological, geophysical and climate evets. Third-party interference, external corrosion, construction defect//material failure significantly outweigh other causes of pipeline failures. According to the EGIG database in Europe, the overall average failure rates for each of the listed causes were 0.020, 0.035 and 0.016 per 1000 km for the period 1970–2022, respectively. With the sharp decline in the number of failures due to the three previously mentioned causes, a fourth cause- ground movements- has emerged as important, with the average annual failure rate of 0.16 per 1000 km over the past five years. It should be noted that Cunha (2016), after analyzing the statistics of pipeline failures from Brazil, Canada, Europe and the United States of America (USA), has concluded that it is reasonable to consider annual failure rate for the past five years in the range of 0.1 to 0.15 failures per 1000 km, with the most common cause of failure being the three causes mentioned earlier.

Once NG is released, different phenomena can occur, such as jet fire, trench fire, fireball and dispersion (Pontiggia et al. 2019). To turn a pipeline failure into a fire, it is necessary for the flammable atmosphere created by the leak to come into contact with an ignition source. This source can be the pipeline failure itself. As the mixture of NG and air can be explosive, dispersion of NG can result in an explosion. This happens when the NG//air mixture is around 5 to 15%.

A significant pipeline failure can result in life loss, personal injury, property destruction and environmental damage, which can create serious threats to communities (Pontiggia et al. 2019). NG pipeline accidents with severe consequences to people, property and the environment that have occurred in Europe in the last two decades include, among others, the explosions in Gislenhien (Belgium: 2004, 23 casualties, 132 injured, significant property damage) (ARIA (Analyze, Recherche et Information sur les Accidents) 2009), Blénod-les-Pont-à-Mousson (France: 2009, 1 casualty) (Chatelet 2019), Janków Podgrodzki (Poland: 2013, 2 casualty, 13 injured, 12 buildings and some part of the forest burned) (Jopek 2016; NIK (Najwyższa Izba Kontroli) 2015), Ludwigshafen (Germany: 2014, 1 casualty, 10 injured, several houses demolished) (DW (Deutsche Welle Broadcast) 2014). Regarding NG pipeline explosions elsewhere in the world, Asuncion (Paraguay: 2004, 250 casualties) (Cheng et al. 2015), San Bruno (USA: 2010, 8 casualties, 53 houses burned down and 120 damaged) (Russo et al. 2014), Kaohsiung (Taiwan: 2014, 32 casualties, 321 injured) (Chen et al. 2020) can be mentioned. The detail description of other NG accidents can be found, for example in Osman et al. (2015) and Sklavounas and Rigas (2006).

The planning and construction of new pipelines, land use planning (LUP) and development in the vicinity of existing pipelines, as well as operating of existing pipelines should be carried out in a way that ensures the safety of the pipeline itself, as well as the protection of local community (people, their property and the environment). In particular, fixed (separation) distances should be maintained from pipelines to residential and other sensitive areas to protect the pipeline and protect these areas against impacts from the pipeline when routing new pipelines and locating new developments near existing pipelines. This important LUP measure is addressed in Article 17 of the C174 ILO (International Labor Organization) Convention (1993) and in two guidelines dedicated to pipeline safety, one issued by the OECD (Organization for Economic Co-operation and Development) (2023) and the other by the UNECE (United Nations Economic Organization for Europe) (2014).

As far as the environmental implications of proposed activities (from projects to plans and policies) is concerned the world’s top instrument considering these matters from 1991 onwards, is the Espoo convention known also as the EIA (Environmental Impact Assessment) convention (UN 1991) together with the SEA (Strategic Environmental Assessment) protocol (UN 2003). The EIA is a comprehensive assessment of the anticipated effects of a proposed project, including those resulting from major accidents and disasters (MADs), on environmental factors, based on which mitigation strategies are proposed. The evolution of the “traditional” EIA with a strong emphasis on biophysical components and minor significance acknowledged to social matters in project assessment (Taylor et al. 2004) towards an “expanded” EIA, in which environmental (related to biophysical//natural factors) and social (including cultural and health) dimensions are equally recognized has resulted to the integration of social impact assessment (SIA) into the EIA framework (Dendena and Corsi 2015; Paterson 2025). The SIA is understood as a process of “analyzing, monitoring and managing the social consequences of development*”* (Vanclay 2003). The “expanded” EIA, which emphasizes a shift of attention from environmental conservation to sustainable development, is often termed the environmental and social impact assessment (ESIA) (Dendena and Corsi 2015; Hughes 1998). The ESIA, with this name attached, is used in countries where the legal definition of the environment is broader and includes biophysical, social, cultural and health aspects. The broader definitions are more common in developing countries (for example in New Zealand, Brazil, the Queensland and New South Wales States in Australia, China, Malaysia and Nigeria) and narrow in developed countries (for example, in the EU (European Union) and MSs (Member States)) (Burdge and Taylor 2012). It is also important to note that even when legislation does not explicitly require the appraisal of social impacts, the range of regulations in place may still apply to social issues (Larsen et al. 2015). The results of the EIA procedure are presented in the EIA//ESIA report (EIAR//ESIAR). In most countries, the EIA procedure is a part of the environmental licensing process required for projects potentially harmful to the environment. Regarding the need for the EIA, for projects involving pipelines transporting hazardous materials, including NG, large pipelines unconditionally require an EIA process, while smaller pipelines when the environmental agency decides to do so, considering the characteristics of the project. Global banks also require a promotor to supply either the EIAR (for projects located in the EU, EFTA (European Free Trade Countries), candidate and potential candidate countries) or the ESIAR (for the projects located in the rest of the world) (Kvam 2024).

Public participation, defined as “the involvement of individuals and groups that are positively or negatively affected by a proposed intervention (i.e., a policy, a plan, a program, a project) subject to a decision making process or are interested in it*”* (Andre et al. 2006), for which the EIA convention (UN 1991) provides a platform, is an essential ingredient for the effective EIA (Boweyer 2023; Glucker et al. 2013). Reference to public participation in EIA (also in SEA) is also found in a number of other international legal documents (Burdet and Sinclair 2024), such as the Aarhus convention (in the EU and its 27 MSs) (UNECE 1998), Escazu agreement (2018) (in Latin America and Caribbean), Article 21 of the Universal declaration of human rights (UN 1948). In the developing countries, their national regulations require that evidence has to be shown in ESIARs how information from the public consultations has been used in the decision-making process (Kantamaturapoj et al. 2018; Lawal et al. 2013; Mwenda et al. 2012; Nadeem and Fischer 2010; Ye et al. 2023). This requirement is also expressed in environmental and social standards for EIARs formulated by global banks (Dendena and Corsi 2015).

Although a vast amount of literature is available in various well-established journals on EIA legislation and practice in different countries, examples of publications exploring the EIA process of NG pipeline projects are rather limited. They cover the following subjects: identification of the optimal pipeline route (Karch et al. 2018; Padash and Artae 2019), the principles of ESIAR preparation (Cekirge et al. 2015), limitations and drawbacks of using preliminary EIAR as an input to environmental licensing (Kirchhoff et al. 2007), addressing the landslide risks in EIARs (Hileman et al. 2021), case studies of risk assessment of MADs within the EIA (Bai et al. 2013; Esford et al. 2004; Jozi et al. 2012; Kalatpoor et al. 2011; Karimi et al. 2014; Kirchhoff and Doberstein 2006; Kwast-Kotlarek et al. 2019; Morgan et al. 1996; Zuñiga-Gutiérrez et al. 2002), the importance of public participation and consideration of social factors in the EIA (Goodland 2006; Lawal et al. 2013; Montaño et al 2021; Ogunlana et al. 2001; Shoobridge and Kapila 1998; 2017), mitigating social conflicts (Boudet et al. 2011).

Still, little is known about the current practice on how MADs are considered in the EIA for NG pipeline projects. This research aims to contribute to the knowledge in this specific area of studying situations in Poland. It is intended to answer the following questions:

- how MADs are considered in EIARs for NG pipeline projects in Poland,
- are any improvements required in this regard, and if so, which ones.

The study combines a legislative survey and a content analysis of a set of Polish EIARs for NG pipeline projects using a qualitative approach. To provide the necessary context for discussing the study’s findings, a review of academic literature (including the publications referenced above) and “grey” literature (i.e., guidelines, EIA reports from other countries) was conducted, identifying two key issues. The first was on approaches of MADs risk assessment in EIA for NG pipeline projects. The second was about the importance of public participation and consideration of social factors in EIA for NG pipeline projects, with a focus on mitigating social conflicts, particularly those fueled by local community concerns about the hazards posed by MADs. The work is considered to provide essential insights for researchers (identifying some research gaps and showing that they deserve attention), EIA practitioners (encouraging them to improve the quality of EIA), decision-makers (showing the importance of public participation in the EIA), regulators (suggesting some legislation improvements) and all other parties interested in the subject (including society and non-governmental organizations (NGOs) who not only have legal rights to participate in the EIA procedure actively, but also whose voice must be taken into account in reforming and updating legislation).

The article consists of six sections, and the following text is structured as follows: Section 2 presents a literature review, Section 3 describes the materials and methods, Section 4 provides the results of the study, and, finally, the discussion and conclusion are to be found in Sections 5 and 6, respectively. The legislation concerning the study is presented in Appendix.

Taking into account that, at the time of this study, the terms “accident”, “major accident”, “disaster”, “risk”, “vulnerability”, “significant environmental effects”, “risk assessment”, “mitigating and monitoring measures”, “social impacts” have not been defined in the context of the EIA Directive, definitions from the EC EIAR guidelines (2017), the IEMA (Institute of Environmental Management and Assessment) primer (2020), the ISO (International Standard Organization) guidelines (ISO 2018; after Fuentes-Bargues et al. 2020) and the IAIA (International Association for Impact Assessment) guideline (Vanclay et al. 2015) have been adopted for this study.

## A Literature Overview

### Approaches for the Risk Assessment of MADs within the EIA, Focusing on Approaches Relating to Pipelines

#### Methods for Assessing the Risk of Major Accidents on Pipelines Adapted for the EIA

It is essential to clarify that special methods of risk assessment dedicated to pipelines have been developed. Most of the methods presented in this chapter address major pipeline accidents (i.e., events such as fires or explosions that cause great damage to the environment and property and loss of life), and only a few directly reference disasters (i.e., man-made/external events (such as excavation works or sabotage) or natural events (such as landslides or floods) with a potential to trigger a major accident). However, as disasters have the potential to cause accidents, they are considered in the methods used to determine the probability of accidents, even if they are not explicitly mentioned.

In general, approaches for assessing the hazard//risk of accidents at hazardous installations, and this also refers to pipelines, can be divided into three categories (LUP guidelines 2006; UNECE 2014):

- generic distance methods: identify the fixed, separation distances between hazardous activity areas and areas for residential, public, or other sensitive activities; and are determined based on the general characteristics of the hazardous activity and the localization,
- consequence-based methods: identify the “worst-credible” potential consequences of accidents and assess the impacts with no clear quantification of the probability of these events,
- risk-based methods: by definition, risk is the probability of a hazard//impact occurring in combination with the consequences//effect on a receptor, should it occur; it is determined either as a numerical value using quantitative risk assessment (QRA) methods or as a descriptive ranking using qualitative or semi-quantitative assessment (SQRA) methods. For QRA methods, two measures are usually considered: individual risk and societal risk. For qualitative and SQRA methods, the outputs are qualitative and assessed using a risk matrix, for example, in a 3×3 risk matrix, the risk is described as high, medium, and low, or using color indicators such as black, grey, and white. These approaches have been developed to support LUP decisions in areas where hazardous installations exist and new developments are planned in their vicinity, or where new hazardous installations are planned.

The various approaches have different advantages and disadvantages. Using the example of an NG pipeline as a representative hazardous installation, this can be described as follows. Although fixed, separation distances between the pipeline and buildings//other structures are easy to implement (Report 281 2004) and the philosophy behind their determination implicitly includes risk evaluation (Fearnehough 1985), this approach does not take into account differences in pipeline design and protection measures used (such as reinforcing the pipeline section with concrete encasement), local environmental conditions (such as ground stability) and population vulnerability (i.e., children and the elderly). The consideration of population density is also simplified (since the so-called location classes determined by the degree of urbanization of an area do not fully reflect the diversity of actual population distribution). The established fixed, separation distances are primarily intended to protect the NG pipeline from damage, rather than the local community. Distances set based on “worst-credible” accident scenarios, on the other hand, are often conservative (Haklar and Dresnack 1999). This means that separation distances determined from special models simulating the effects of pipeline failure are often very large, which can result in unjustified loss of development opportunities in a given area. Risk-based approaches potentially allow more rational and efficient use of land around the NG pipelines (Souka and Tsakiris 2016), but they are generally more complex, costly and time-consuming. QRA methods, in particular, not only require access to extensive data and sophisticated risk assessment tools, but also the expertise to perform complex risk assessment calculations. It should also be added that, their results can be highly dependent on the assessment tools, input data, assumptions and risk assessment criteria (Dawotola et al. 2012; Hopkins et al. 2011). SQRA methods appear to be a reasonable solution. They are less complex than QRA methods, but still take into account variability in local environmental and technical conditions and population-specific factors (GESIP 2014). The ability to take into account population density and population vulnerability are important arguments for using risk-based methods, since in an urban area the same NG pipeline accident can cause significantly more casualties and damage than in a rural area.

Regarding the application of the hazard//risk assessment approaches in LUP decisions for NG pipelines, the combination of generic distances and the QRA method has become common practice in the UK (Goodfellow and Haswell 2006; Graham et al. 2008; Haswell et al. 2009) and Hong Kong (EMSD (Electrical and Mechanical Services Department) 2021), and was approved in the Netherlands, among others (Kooi et al. 2015). In 2014, a combination of the consequence-based approach and the SQRA method developed by GESIP (Groupe d'études de sécurité des industries pétrolières) was approved in France (Chatelet 2019; GESIP 2014; Descourriere and Chaumette 2006) as a replacement for the consequence-based approach.

Research on the development of new QRA methods for effective LUP in the vicinity of existing pipelines and for new pipeline projects have been carried out in Canada (Henselwood and Phillips 2006), China (Ma et al. 2013; Yin et al. 2022), Italy (Vianello and Maschio 2014) and Korea (Jo and Ahn 2002). Henselwood and Phillips (2006) simplified the risk assessment calculations proposing a collection of matrices. This matrix- based approach allowed to give a measure of risk at both an NG system and across the system. Ma et al. (2013) constructed a QRA system for urban NG pipeline networks enhancing the QRA method by applying the GIS (Geographic Information System). Vianello and Maschio (2014) proposed the QRA methodology for the national NG distribution networks, adapting solutions described in the literature for risk identification, estimation of failure frequency and estimation of consequences. To display the damage zones for each section of the pipeline they employed the ArcGIS software and “Buffer Wizard” tool. Yin et al. (2022) improved the QRA method by establishing two models: a failure probability model using improved historical accident and disaster probabilities, and a risk consequence model that incorporates direct and indirect losses. Jo and Ahn (2002) proposed a simplified QRA method for an NG pipeline by introducing two parameters: “a fatal length” (defined as “a weighted length of pipeline within which an accident has the fatal effect on the person at a specified location”) and a “cumulative fatal length” (defined as “the length of pipeline in which an accident results in N or more fatalities”). These parameters were estimated directly within the GIS.

Regarding applying these methods in the EIA process of NG pipeline projects, Kirchhoff and Doberstein (2006) discussed the QRA method for assessing the risk related to both the realized and alternative NG pipeline trajectories in the Sao Paolo State, Brazil. They carried out calculations for critical segments of the pipeline (i.e., where the pipeline crossed near or directly under populated areas), for which an optional route was suggested, and compared the risk related to both routes. They found that the risk acceptance criteria for pipelines applied by the Sao Paolo State were too liberal compared to other criteria used worldwide (for example the individual risk levels in this State were defined as acceptable when less than 1 × 10^-5^ per year, while in the UK, Western Australia, Venezuela they had to be less than 1 × 10^-6^ per year, and in the Netherland still 100 times stricter). They suggested that the criteria be strengthened to adjust them to international standards, which would allow for greater safety for those people living in the vicinity of the new NG development. Concerning the risk of pipeline accidents, this study focused on protecting people, i.e., on risk assessment for human health.

Also, the EIA process of the high-pressure NG pipeline section from Wierzchowice to Kiełczów, Poland, as reported by Kwast-Kotlarek et al. (2019), focused on the safety of people. They stated that the results of QRA studies by Jo and Ahn (2002), Jo et al. (2004), Jo and Crowl (2008) were referred to in the EIAR. These results showed that in the event of an accidental release and ignition of NG, the hazardous zone ranged from less than 20 m for the smaller, lower-pressure pipeline to more than 300 m for the larger, higher-pressure pipeline.

In addition to the three risk assessment approaches, which mainly focused on the effects on people and were used to support LUP decisions, another group of risk assessment methods needs to be introduced here, i.e., indexing (scoring) methods. These methods only allow a relative assessment of risk along the pipeline. They use quantitative or qualitative input data to produce quantitative outputs with a scoring algorithm. The scores assigned to the inputs are combined to obtain a quantitative index score with no units. The most popular method of combining inputs and deriving scores is to sum individual, and in some cases weighted, risk factor scores. Substantial differences exist between the index models in terms of the specific input variables that are considered in the risk quantification, the way the scores are assigned to these variables, the way the scores are weighted, and the way the weighted scores are combined to produce an overall index.

Most indexing methods were initially developed and have been in wide use, underpinning numerous pipeline integrity and risk management activities in the industry. Every pipeline section is assessed considering all of its attributes. Individual pipeline sections are then ranked by their relative risk scores to set a priority for maintenance, monitoring and other risk-mitigating actions. The most used indexing method is that presented in Muhlbauer’s manual (2004). Muhlbauer’s original method is used for assigning numerical scores to each of four indices of hazard: “Corrosion”, “Design”, “Third Party Damage” and “Incorrect Operations”. These factors are then combined numerically, and a “Leak Impact Factor” (i.e., a consequence factor, which includes “Product Hazard” and “Dispersion Parameter”) is used to provide a relative measure of pipeline risk. A value for each hazard index is calculated by collecting relevant information about the pipeline and its surroundings that is considered to increase or reduce pipeline safety. The original Muhlbauer’s method is focused on public safety. The system can be developed to account for threats to pipeline personnel and sensitive environmental elements.

Esford et al. (2004) modified Muhlbauer’s method to address geohazard more fully, creating a comprehensive set of geohazard risk variables and assigning weights so that the risk could be assessed more accurately and objectively. They divided “Geohazards” into three broad categories: tectonic, geotechnical and hydrotechnical hazards. They applied this method to determine the risk for an operating pipeline transporting crude oil and batches of LPG between Santa Cruz and Cochabamba, Bolivia.

Bai et al. (2013) modified the original Muhlbauer’s method by introducing a three-dimensional risk matrix for urban NG pipelines in China. The horizontal axis in the matrix represents fault frequency, and the vertical axis represents consequences.

Regarding the application of Muhlbauer’s indexing method or its modifications in the EIA process, a literature review revealed three studies on the risk assessment of NG pipeline projects conducted in Iran (Jozi et al. 2012; Kalatpoor et al. 2011; Karimi et al. 2014). Karimi et al. (2014), performing an EIA study of the Ahar-Duzduzan NG transmission pipeline applying a matrix method (in which the matrix for the construction phase included 19 micro activities against 12 environmental factors and the second matrix for the operational phase evaluated 15 micro activities against 15 environmental factors), recommended the Muhlbauer’s indexing method for assessing the risk of pipeline accidents. They did not specify what version of the Muhlbauer’s method was used in their study. Kalatpoor et al. (2011) modified Muhlbauer’s indexing method when assessing the public safety, occupational health and environmental risk of an NG transmission pipeline in the Gachsaran area. The difference was that they used ALOHA software instead of Muhlbauer’s pattern to assess the consequences. Even though they included more parameters in the “Leak Impact Factor” than in the original Muhlbauer’s method (i.e., “Target”, “Population Density”, “High-Value Area”, “Product Hazards”, “Acute Hazards”, “Chronic Hazards”, “Threat Area”), they still acknowledged that their method was more credible for occupational safety and health than environmental issues. Jozi et al. (2012) used an indexing method with an analytical hierarchy process to assess the risk to people and the environment of the Aabpar–Zanjan NG pipeline. They used the analytical hierarchy process to evaluate factors due to differences in the total effective level of these factors. They used four hazard indices from the original Muhlbauer’s method but developed the “Leak Factor” by adding the third parameter, i.e., “Ecological Sensitivity”. In these EIA studies, the risk assessment was carried out along the pipeline route using GIS software.

Zuñiga-Gutiérrez et al. (2002) proposed and applied a methodology involving indexing methods to evaluate alternative routes for the NG pipeline in the Campeche State, Mexico. They modified Yapp’s indexing method, which was initially used for highways (Spellberg 1992) for the ecological assessment of alternative routes. The environmental damage resulting from the construction of the investment was considered in relation to the ecological importance of the habitats along the path of the linear investment and its length. They developed an original indexing method for assessing the risk of accidents for both routes. They considered the potential for damage to the pipeline caused by human behavior, agricultural activities, the location of inflexion points, and topography along the route. Regarding the risk of pipeline accidents, this study focused on protecting the pipeline rather than the public, specifically assessing the project’s vulnerability to accidents.

Morgan et al. (1996) also carried out a study in which risk assessment for people (human safety risk) and the environment (environmental risk) of gasoline transmission pipelines was performed separately using different methods. They assessed human safety risk using the QRA method, in which the probability and consequences of pipeline failure were combined to produce transects of individual risk and FN curves of social risk (i.e., Frequency and N or more fatalities) and environmental risk using the original scoring method, which considered that pipeline traversed very different areas in terms of habitat, land use, water courses, etc. An overall measure of environmental risk was produced for each pipeline segment, combining the index of environmental consequences with the leak frequency of either deep- or shallow-buried pipes. The results were presented using MapInfo software, where the safety risk was indicated by different colors on the pipeline itself, and the relative environmental risk was indicated by coloring the corridor centered on the pipeline, allowing areas of high environmental and safety risk to be immediately identified. In both cases, four risk categories were established.

GESIP developed another scoring method for environmental risk assessment of pipelines transporting hazardous materials. This method and the SQRA method for human risk assessment (mentioned at the beginning of this section) are described in the GESIP guidelines (2014). It focuses on the potential impact of the transported products on surface water, groundwater, soil and protected or recognized natural areas. This method assumes that the potential severity of the spill depends on the nature and volume of the released substance, the nature of the terrain, the distance to the target, and the nature of the target. Three environmental severity classes have been introduced: severity class of 0 (no measures are necessary), class of 1 (a level 1 measure is required), and class of 2 (a level 2 measure or a combination of two level 1 measures is required). These measures exist or are to be implemented and are of two types: prevention and intervention. It was decided not to focus on the effects on the atmosphere, which, as the guidelines state, are difficult to quantify. According to the GESIP method, “gaseous products, including NG, are unlikely to contaminate soil or water; therefore, their effects are not examined as part of this environmental analysis”.

#### Approaches of the Risk Assessment of MADs Adapted for the EIA, Initially Developed for Other Applications

Another approach to environmental risk assessment using the “source>pathway>receptor” concept is described in the CDOIF (Chemicals and Downstream Oil Industries Forum) guidance (2016) for Seveso sites in the UK (i.e., the establishments included in the Directive 2012/18/EU (2012), also known as the Seveso III Directive). In the absence of specific EIA methodological guidance on environmental risk assessment, it was applied in the EIA process of the British Southampton to London [aviation fuel] pipeline project (EIAR 2019d), and it is included in this review for its potential and originality. The CDOIF guidance (2016) starts by defining the types of harm to be covered in an environmental risk assessment and the way in which the harm ought to be characterized for assessment purposes. The level of environmental harm regarded as serious (i.e., a MATTE event, “Major Accident To The Environment”) for different receptor types is defined in combination with extent (distance/area), severity (degree of harm in the area of impact) and duration (period of recovery). For environmental harm regarded as being serious, all parameters are required to be above the receptor thresholds specified in the guidelines. Four environmental receptor types are included: groundwater bodies, marine habitats, freshwater habitats and terrestrial habitats, as well as the built heritage environment.

The assessment process includes the following stages (Marsh-Patrick et al. 2020): identification of relevant MATTE events for the installation, estimation of the failure rate taking into account different failure scenarios, assessment of the likely severity of the impact on selected environmental receptors and the estimated duration for which the receptor may be affected (usually the unmitigated frequency is assessed initially), accounting for the benefit mitigation measures, that reduced the duration or severity of the environmental harm, and finally determination of risk using the CODIF grid, according to which the risk is classified as tolerable, tolerable if ALARP (“As low as reasonably practical”) or intolerable, which in turn indicates what (if any) improvements are needed to ensure the risk is reduced to an accepted limit. The ALARP principle is used in risk management as required by the UK HSE (Health and Safety Executive) regulations. The guidance notes that quantitative environmental impact assessment entails substantial uncertainty and complexity. While some quantification is likely possible, a significant level of qualitative judgment is required. As far as NG is concerned, the guidelines state that it has no environmental hazard classification and is cited in SDSs (Safety Data Sheets) as “no ecological damage caused by this product”. Hence, it is reasonable to assume that there is no MATTE potential. However, exclusion can only be confirmed after considering the “Extremely flammable hazard”(e.g., potential for explosion impact leading to MATTE to the built environment).

An application of the CDOIF guidance (2016) in the EIA context can be found, for example, in the UK EIAR concerning the assessment of the Southampton to London [fuel aviation] pipeline project (EIAR 2019d).

Fuentes-Bargues et al. (2020) recommend five out of 45 risk assessment techniques defined in the IEC (International Electronic Commission) guidelines (2019) for the analysis of project’s vulnerability to MADs within the EIA process i.e., “Cause and Effect Analysis”, “Consequence//Probability Matrix”, “Failure Mode and Effects Analysis” (FMEA), “Scenario Analysis”, and “Structured What If Technique” (SWIFT) techniques. The last method, combining various semi-quantitative and qualitative classifications of the probability of hazards//impacts and their consequences//effects, allows the determination of risk either numerically or qualitatively. Its application to the EIA process in Slovakia can be found in Zeleňáková et al. (2017; 2020). Irish EIA practitioners often use this technique, adapting the criteria to classify the risk from a selection of sources, for example, from the two risk assessment guidelines one prepared by the Department of Environment, Heritage and Local Government (DoEHLG 2010) and the other by the Department of Defense (DoD 2017). Zeleňáková et al. (2020) indicated that the “Consequence//Probability Matrix” technique could be extended to other countries, taking into account national realities, standards, and regulations.

#### Methodology Dedicated to the Risk Assessment of MADs within the EIA

In 2020, the IEMA (Institute of Environmental Management and Assessment) published a primer, “Major accidents and disasters”, presenting a risk assessment (using a “source>pathway>receptor” concept) based on known practice in the UK. The methodology set out in this document follows three stages: screening, scoping and assessment. The screening stage aims to identify whether the project is vulnerable to Major Accidents and Disasters (MADs) and considers whether it could potentially result in significant adverse effects. The scoping stage provides a more detailed assessment of the potential for significant effects resulting from MADs associated with the project. According to the IEMA primer (2020) the MADs can be excluded from the assessment when it can be clearly proven that for a given hazard, there is no “source>pathway>receptor” linkage capable of causing a MAD or potentially leading to a significant environmental effect, or all possible MADs are properly addressed elsewhere [i.e., in other chapters of the report] or covered by implemented design measures or compliant with both legislation and best practices. As a result, events with high probability and high consequences (unacceptable for any project are managed or designed out of the development) and events with low probability and low consequence (and thus not significant) are scoped out. The assessment tends to focus on events with a low probability but potentially high consequences (i.e., major spills, explosions, fires, etc., where significant environmental effects are likely). Those that existing design measures and standards cannot mitigate are scoped into the assessment. This stage gives an additional insight into the probability of the identified hazard, its consequences, and the requirement for additional mitigation.

To define the level of risk to people and the environment, the following factors can be included: extent, duration, severity of the effect, receptor sensitivity and effect required to restore the affected environment. Without specific significance criteria for MADs, the significance was based on criteria for notification of major accident hazards from Annex VI of the Seveso III Directive transposed to the UK regulations. As a result, the significance threshold for a new project has been set at a level that results in lost life, lasting injury or environmental receptor damage. If the risk cannot be described as ALARP, additional mitigation is required to reduce it to ALARP levels. The primer does not require a stand-alone risk assessment for MADs, as existing public sources in the UK are available to inform the baseline risk level. The primer was developed with the aim of generating comments and some discussion on which future guidance could evolve.

The methodology from the IEMA primer (2020) was applied, for example, in the UK EIA process carried out for the Keadby 3 CCS [carbon capture and storage] gas power station project (EIAR 2023) and in the Irish EIA process for the Viking CCS pipeline project (EIAR 2023). The practice showed that the risk-based assessment of MADs is usually presented in a tabular format.

### The Relevance of Public Participation and Consideration of Social Factors in EIA for NG Pipeline Projects, with a Focus on Mitigating Social Conflicts, Particularly those Fueled by Local Community Concerns about the Hazards Posed by MADs

According to Aureli and de Wall (2000; after Bergmuller 2015) conflicts arise “when individuals act on competing goals and interests”. Energy infrastructure projects, including natural gas (NG) pipeline projects, have caused various conflicts between stakeholders, particularly between the local community in the project area and the project proponent (Bergmuller^ 2015^; CBI and ERM Report 2020).

Opposition to the siting of a pipeline can be based on several reasons, of which social impacts related the hazard posed by MADs, such as concerns about public health and safety, fear of damage to the landowner property or other material assets and natural and cultural environment as a result of MADs (Boudet and Ortolano 2010; Boudet et al. 2011;CBI and ERM Report 2020) are important contributors. According to the IAIA guideline (Vanclay et al. 2015), the social impacts related to the hazard posed by MADs fall within three out of eight social impact categories: “people’s health and well-being”, “their environment”, and “their fears and aspirations”.

Studies carried out by different researchers showed that, in addition to careful project design (Boudet et al. 2011), effective public participation (in particular the participation of those affected by the project) (Shoobridge and Kapila 1998), properly addressing concerns of the public (Ogunlana et al. 2001), adequately assessing social impacts for the commune, presenting the mitigation measures for the negative ones and demonstrating benefits for the commune that the project can deliver (Prenzel and Vanclay 2014), improving understanding during the pipeline project implementation (CBI and ERM Report 2020) helps to build stakeholder support for project implementation and can considerably contribute to de-escalate, mitigate or even avoid the possible social conflicts.

## Materials and Methods

The study involved two steps. First, the Polish EIA regulations were reviewed in relation to the EU law and then a content analysis of selected Polish EIARs for NG pipeline projects was carried out regarding the consideration of MADs. Regarding the reviewed Polish EIA regulations (Table 1), they cover the period in which the analyzed EIARs were prepared with time margins, i.e., from 1 of November 2008, when the Act 2008/199/1227) (2008; hereafter referred to as the 2008 EIA Act) became effective, to the present. As the transposition of the EIA Directive 2014/52/EU (2014; hereafter referred to as the 2014 EIA Directive) took place in Poland in 2015 by the Act 2015/1936 (2015), the resulting amended version of the Polish EIA Act is hereafter referred to as the 2015 EIA Act. It is important to add that after this date, the amendments to the 2015 EIA Act did not cover the MADs issue. In this study eight EIARs for NG pipeline projects were analyzed (Table 2). These are the only complete reports available to the author. They were obtained by searching websites using keywords such as “EIAR” and “natural gas pipeline” (expressed in Polish). It should be noted that EIARs for NG pipeline projects are rarely available online. The reports presented were developed between 2009 and 2021. In total, the author has analyzed close to 3200 pages.

**Table 1:** Main Polish regulations relevant to the study included in main acts and ordinances covering the period of 2008–2021 with time margins

Binding period	Regulations and their Evolution in Time	Issues in the Act/Ordinance Referred to in the Subsection on “Polish Legal Regulations Regarding the Consideration of MADs in the EIA in Relation to the EU law“
Acts concerning the dissemination of information on the environment and its protection, public participation in environmental protection and environmental impact assessment		
15.11.2008- till now	Act 2008/199/1227 (2008)	MADs risk assessment, public participation, a social dimension of the project’s impact, social conflicts
01.01.2017- till now	Act 2015/1936 (2015) upgrading the 2008 EIA Act. It resulted in 2015 EIA Act	as above
Regulations concerning the determination of the types of projects likely to have a significant impact on the environment and the detailed conditions connected with qualifying a project to draw up a report on environmental impact		
08.12.2004-15.11.2010	Ordinance 2004/257/2573 (2004)	classification of projects
08.06.2005-15.11.2010	Ordinance 2005/92/769 (2005) upgrading the Ordinance 2004/257/2573 (2004)	as above
31.08.2007-15.11.2010	Ordinance 2007/158/1105 (2007) upgrading the Ordinance 2005/92/769 (2005)	as above
15.11.2010-11.10.2019	Ordinance 2010/213/1397 (2010b)	as above
01.08.2013-11.10.2019	Ordinance 2013/817 (2013b)	as above
10.09.2019-11.10.2019	Ordinance 2019/1839 (2019)	as above
Regulations concerning the technical requirements to be met by gas networks and their location		
12.12.2001-05.09.2013	Ordinance 2001/97/1055 (2001a)	minimum distances from the NG pipeline to buildings and other structures, a control zone, location classes
05.09.2013- till now	Ordinance 2013/640 (2013a)	as above
Regulations concerning the minimum health and safety requirements relating to the possibility of an explosive atmosphere in the workplace		
22.07.2006-31.10.2010	Ordinance 2006/121/836 (2006)	explosive atmospheres classes
31.10.2010-till now	Ordinance 2010/138/931 (2010a)	as above
Acts concerning spatial planning and land use management		
27.05.2021-till now	Act 2021/922 (2021) upgrading the Act 2003/80/717 (2003)	a consultation zone

**Table 2:** EIARs Included in the Study

1. Names of Nodes Delimiting NG Pipeline Section 2. Pipeline Characteristic (material/diameter/ maximum operational pressure/section length)	Location (voivodship: commune)	Reference
1. Swinoujście-Goleniów 2. Steel/800 mm/10.0 MPa/68.8 km	Zachodnio- pomorskie: Goleniów, Międzyzdroje, Stępnica, Świnoujście, Wolin	EIAR 2009
1. Hermanowice- Strachocina 2. Steel/700 mm/8.4 MPa/71.9 km	Podkarpackie: Bircza, Dydnia, Fredropol, Przemyśl, Sanok, Tyrawa Wolowska, Ustrzyki Dolne	EIAR 2011
1. Strachocina-Polish border 2. Steel/1000 mm/8.4 MPa/59 km	Podkarpackie: Bukowsko, Komańcza, Sanok	EIAR 2016
1. Gustorzyń-Lesniewice 2. Steel/1000 mm/8.4 MPa/51.4 km	Kujawsko-pomorskie: Baruchowo, Brześć Kujawski, Choceń, Włocławek; Mazowieckie: Gostynin	EIAR 2019a
1. Leśniewice- Rawa Mazowiecka 2. Steel/1000 mm/8.4 MPa/100 km	Łódzkie: Bedlno, Oporów, Głuchów, Godzianów, Łowicz, Łyszkowice, Maków, Nowy Kawęczyn, Rawa Mazowiecka, Skierniewice, Zduny, Żychlin; Mazowieckie: Gostynin, Pacyna, Szczawin Kościelny	EIAR 2019b
1. Rawa Mazowiecka-Wronów 2. Steel/1000 mm/8.4 MPa/154 km	Mazowieckie: Jedlińsk, Głowaczów, Gniewoszów, Kozienice, Mogielnica, Radzanów, Sieciechów, Stara Błotnica, Wyśmierzyce; Lubelskie: Końskowola, Puławy, Zyrzyn; Lódzkie: Rawa Mazowiecka, Regonów, Sadkowice	EIAR 2019c
1. Oświecim-Tworzeń 2. Steel/1000 mm/8.4 MPa/44 km	Małopolskie: Bukowno, city of Jaworzno, Chrzanów, Libiąż, Chełmek, city of Oświęcim; Sląskie: Sławków,	EIAR 2020b
1. FSRU terminal-barrage unit 2. Steel/1000 mm/8.4 MPa/40 km	Pomorskie: city of Gdańsk, Pruszcz Gdański	EIAR 2021

The review of the legislation (Appendix 1) was carried out with the method of legal interpretation, i.e., determining the meaning of the content of a provision. In analyzing the content of EIARs, the author was inspired by the qualitative method of Mayring (2000; 2014). A review of the legislation allowed to select specific issues related to MADs, which were used to identify key analytical categories. These “first choice” analytical categories were reviewed and adapted to the content of the EIARs. In this way, the analytical categories used to examine the content of EIARs take into account both the legal framework and the specifics of the documents examined. The analytical categories used and their description are given in Table 3. Text analysis was an iterative process. It involved carefully reading each EIAR document, marking passages in the text that the author identified as relevant to a specific issue (i.e., meeting the category description), noting the category label and taking research notes. The author conducted the analysis individually. To minimize the lack of intersubjective verification, which is a methodical limitation of analysis with a single coder, the author repeated the coding procedure and carefully compared the results from the retest, checking for consistency. The results of the content analysis of the EIARs were presented for each document separately in a table using graphical symbols (Table 4 in the “Results of the study: Analysis of the set of EIARs in terms of the consideration of MADs” section) and summarized for the whole set of documents descriptively. This method was used by the author in two other similar studies, which aimed to investigate how risks to people and the environment from accidents at hazardous facilities and pipelines were addressed in land use management plans and SEA reports in Poland (Markiewicz 2020; 2023). It should be emphasized that although the EIA is based on the same principles as the SEA, i.e., in both processes, the focus is on environmental, social and economic considerations, the EIA process is less strategic and more detailed than the SEA.

**Table 3:** Analytical Categories Applied in the Content Analysis of EIARs and their Description

Analytical Category	Label	Description
Control zone	C1	Providing information on the width of the control zone and restrictions//prohibitions on land use in its vicinity.
Explosion hazardous zone	C2	Providing information on the extend of the zones where explosive atmospheres may occur (in meters, on the drawing, indicating that it extends to the fence surrounding above-ground equipment), mentioned or described in more detail.
Minimum distances	C3	Reporting the minimum distances from the NG pipeline to houses//other buildings in detail along the entire section of the pipeline or only for the selected buildings closest to the pipeline (in meters or in descriptive form), with an assessment of whether the required minimum distances have been maintained.
Location of protection//other areas	C4	Identification of nature protection areas, water intake protection zones, archeological sites, historical monuments, other sensitive areas (in meters or in a descriptive form); listed or described in more detail.
Natural and man-made disasters	C5	Reporting the possibility of natural disasters (such as landslides, flooding, others) or those related to human activity (such as construction disasters, third party interference, others) that may cause damage to the NG pipeline (listed or described in detail).
Definition of “major accident” and “ major industrial accident”	C6	Providing definition of the listed terms that are in the Polish legal system.
Identification of types of accidents on NG pipelines	C7	Presenting the possible types of accidents (fire, explosion, dispersion); mentioned or described in more detail.
Probability and consequences of MADs	C8	Presenting the frequency of occurrence//probability and consequences//effects of MADs, their classification by probabilities and consequences.
Definition of “risk”	C9	Providing definition of the term that is in the Polish legal system.
Risk assessment	C10	Reporting the results of the risk assessment (defined as above) carried out as a part of the EIA process or using other sources.
Project’s impact in case of MADs on surroundings	C11	Providing a description of impact of MADs on the population (including human health and living conditions) [natural] environment (including climate), material assets, monuments, landscape (including cultural landscape), availability of deposits.
Mitigation measures	C12	Presenting technical and organizational solutions (by listing or describing in detail) (such as insulating coatings, cathode protection system, automatic shut-off of a given section, others) to prevent or reduce the probability//effect of a failure (listed or described in detail).
Monitoring measures	C13	Presenting how the operation of the pipeline will be monitored.
Preparedness and response to emergencies	C14	Presenting the actions planned in case of an emergency (listed or described in more detail).
Potential social conflicts	C15	Presenting the causes of possible social conflicts (such as the concerns of the local community about possible pipeline-related accidents, deterioration of living conditions and//or the environment, destruction of a plot) and ways to mitigate them by choosing the option suggested by the local authorities and benefits for the municipality.
Communication with the public	C16	Providing information on the investor’s communication plan with the public (supporting the activities of the authority conducting the procedure for the issuing a decision on environmental conditions (DEC) obliged to conduct information and consultation procedures), reporting on the activities carried out and methods to minimize potential social conflicts.

**Table 4:** Results of Content Analysis of the EIARs

EIAR nr1	EIAR nr 2	EIAR nr 3	EIAR nr 4	EIAR nr 5	EIAR nr 6	EIAR nr 7	EIAR nr 8
Names of junctions defining a section of an NG pipeline							
Swinoujście-Goleniów	Hermanowice- Strachocina	Starchocina-Polish border	Gustorzyń-Lesniewice	Leśniewice- Rawa Mazowiecka	Rawa Mazowiecka-Wronów	Oświecim-Tworzeń	FSRU terminal barrage unit
GENERAL INFORMATION							
Development of the report (year)/issuing the DEC by the locally competent RDEP							
2009/2009	2011/2011	2016/2017	2019a/2019	2019b/2020	2019c/2020	2020/2021	2021/2021
The project is within the sector of liquefied NG regasification terminal investments (T); it was made subject to systematic assessment (R) as a rule, by the authority decision (D); Number of considered investment variants							
T/R/3	T/D/2	T/R/2	T/R/2	T/R/2	T/R/2	T/R/2	T/D/3
Number of pages in the chapter concerning accidents(and) disasters; social conflicts;in the whole report							
3/3/383	4/3/351	3/3/452	15/3/344	3/2/493	18/2/428	3/4/387	3/1/304
CATEGORIES							
C1: Control zone (C) determined: in meters (m); limitations//prohibitions (R) in it specified							
C:12 m/R	C:12 m/R	C:12 m/R	C:12 m/R	C:12 m/R	C:12 m/R	C:12 m/R	C:12 m/R
C2: Explosion hazardous zones (Z) for the above-ground facilities assessed: in meters (m), shown on a graph (s), is within the fence of the facility (f); detail description (!); N means Z not assessed							
Z:l	Z:m!	Z:dl	Z:m!	Z:m	Z:l!	Z:m	N
C3: Minimum distances from the pipeline to the houses (H)//other buildings (O) determined: by description (d), in meters (m); very detail data (!); the DMIN: maintained (*), not maintained in some places (^<^);							
H^+^:m ^<^	HO^+^:m^<^!	H^+^:dm*!	H^+^:dm*!	HO^+^:d^1^	HO^+^:d^2^!	H^+^:d^2^!	H^+^:d^2^
C4: Location of natural protection areas (N), protection zones of water intakes (W), archaeological sides (A), monuments (M), other sensitive areas (O) given: by description (d), in meters (m); very detailed data (!)							
NWAM!md	NWAM!md	NWAM:md	NWAM!md	NWAM!md	NWAM!md	NWAMO!md	NWAMO!md
C5: Information on the possibility of and potential harm to the pipeline due to landslide (L), flooding (F) other natural disasters (N), construction disasters (C), other disasters such as third-person activity (M) given; described in detail (!)							
M	LF	LFN!	LFNCM!	LFN	LFNCM!	LFNCM	LFNCM
C6: Major accident and major industrial accident definitions given (Y)							
Y	Y	Y	Y	Y	Y	Y	Y
C7: Identification of types of accidents on NG pipelines: fire (f), explosion (e), dispersion (d) provided; in detail (!)							
C8: Information on frequency//probability (p) and consequences (c) of accidents (A)//disasters (D) given; described in detail (!); their classification (C) by probability//consequences given (Y)/(Y), not given (N)/(N)							
A:pc; C:N/N	A:pc; C:N/N	A:c; C:N/N	A:p!c!/D:p; C:N/N	A:pc; C:N/N	A:p!c!/D:p; C:N/N	A:p/D; C:N/N	A:p!c!/D; C:N/N
C9: Risk as the probability of hazards in combination with their consequences defined (Y), not defined (N)							
N	N	N	N	N	N	N	N
C10: Risk as the probability of hazards in combination with their consequences assessed (Y), not assessed (N)							
N	N	N	N	N	N	N	N
C11: Project’s impacts in case of accidents (A) and disasters (D) to population (including human health and living conditions) (p), the [natural] environment (including climate) (e), material assets (a), monuments (m), landscape (including cultural landscape) (l), availability of deposits (d) are discussed.							
A:pe	A:pe	A:pe	A:pe/D:pe	A:pe	A:pe/D:pe	A:pe	A:pe/D:pe
C12: Mitigation measures such as insulating coatings (I), cathodic protection system (C), automatic cut-off of a given section (A), other (O) measures proposed; described in detail (!)							
ICA	ICA	ICAO	ICAO!	CO	ICAO!	ICAO!	ICO
C13: Monitoring measures (M) proposed; described in detail (!)							
M	M	M	M!	M	M!	M!	M
C14: Information on preparedness and response to emergencies (R) provided; described in detail (!)							
R	R	R!	R!	R	R!	R	
C15: Causes of possible conflicts (C) such as concerns of the local community about possible accidents on the gas pipeline(a), deterioration of living conditions (l) and the environment (e), damage to plots (p), adoption of a location variant other than the one suggested by the local authorities (v) are given/ Benefits for the community specified (B)							
C:acep/B	C:acep/B	C:acep/B	C:acep/B	C:acep/B	C:v/B	C:acep/B	C:acep/B
C16: Information on the investor’s plan for communication with the public (P) (supporting the activities of the authority conducting the procedure for issuing a DEC obliged to conduct the proceedings to inform and consult the public) presented/some of activities reported (R)//methods of minimizing the possible social conflicts (M) given							
M	M	P/M	P/M	M/R	P/M/R	P/R	P/R

## Results of the Study: Analysis of the Set of EIARs in Terms of the Consideration of MADs

To answer the question regarding the consideration of MADs in the Polish EIARs, sixteen detailed issues (analytical categories) labeled as C1 to C16 (Table 3 in the “Materials and methods” section) were used. The detailed results of the content analysis of the eight EIARs, proceeded by information on the name of the junctions defining the section of the planned NG pipeline and the type of the project, the dates of development of the EIAR and issuing the DEC for the project, the number of investment variants, are presented in Table 4. They can be summarized as follows:

1. All the projects were within the sector of liquefied NG regasification terminal investments, so the locally competent RDEP issued the DEC. Six projects were subject to the EIA as a rule, and two projects were subject to the EIA by the authority’s decision.
2. Three EIARs, which were produced prior to 1 of January 2017, i.e., before the 2015 EIA Act came into force, briefly discuss the issue of accidents, primarily in a separate chapter usually entitled “Major industrial accidents potential”. All other EIARs describe the issue of accidents and natural, construction, and other man-made disasters in one or even three separate subchapters, but only in two reports is the description detailed. The issue of accidents//disasters is also referred to in other sections of the reports. All EIARs included the chapter analyzing the possibility of social conflicts.
3. All eight EIARs state that the pipeline is routed mainly through areas which, according to the Polish regulations, belong to the location class III or II. Seven reports specify the distances from the pipeline to the nearest buildings along the pipeline route, four of which give the distances numerically. In one report, the information is specified in an appendix that is not available on the Internet, so it is not known whether the distances are given numerically. Data in two reports indicate that DMINs are not met in some places (assigning the sites to the location class II). In three other reports, it is stated several times that the pipeline route crosses the areas designated as residential in the local management plans. All reports describe in detail the location of other sensitive areas in the vicinity of the pipeline route and specify the distances to the nearest of these or the length of the pipeline segment crossing the sensitive terrain.
4. All EIARs include information on the control zone width in the linear part of the NG pipeline and the limitations//prohibitions therein. The extent of the explosion hazardous zones for above-ground facilities is reported in seven reports, but only in four cases are their radii given numerically. In the other three cases, it is stated that the extension of the zone is within the facility’s fence.
5. Nearly all EIARs (except the one from 2009) provide information on the possibility of and potential harm to the pipeline due to natural disasters. Four documents out of five prepared after 1 of January 2017 provide information referring to natural, construction, and other man-made disasters. Still, only two reports from 2019 discuss natural, construction, and other man-made disasters in detail. None of the reports provides information on the location of Seveso establishments near the pipeline that would pose a major accident hazard.
6. All EIARs explain definitions of “major accident” and “major industrial accident” from the Polish legal system and identify types of potential accidental phenomena. However, all reports discuss the frequency//probability of accidents and their consequences to people and the [natural] environment, but only three EIARs out of five prepared after 1 of January 2017 report extended pipeline accident statistics and describe possible consequences in detail. All reports state that, however, pipeline accidents may generate negative consequences to population (including human health and living conditions), the [natural] environment and infrastructure (of which some can be serious), such emergency situations are very improbable. No classification of probability of accidents and no classification of their consequence is provided. The risk, defined as the probability of hazards//impacts in combination with their consequences//effects, was not assessed within any of the EIA processes studied, and none of the reports refers to specific results of risk analysis from other sources.
7. All EIARs provide information on mitigation and monitoring measures, and details on emergency preparedness and response, but only three EIARs out of five prepared after 1 of January 2017 discuss these in detail.
8. All EIARs analyze causes of possible social conflicts and point out concerns of the local community about possible accidents on the NG pipeline, deterioration of living conditions and the [natural] environment, damage to plots, adoption of a location variant other than the one suggested by the local authorities. They provide information on benefits for the local commune resulting from the construction of the project.
9. Information on the investor’s communication plan with the public, which is carried out to strengthen the mandatory procedure of informing and consulting the public carried out by the authority (Regional Director of Environmental Protection, RDEP) conducting the decision on environmental conditions (ECD), is provided in the EIARs, and/or methods of minimizing the possible social conflicts are listed. Four reports inform on the course of these activities.

1. Detailed information on the development in the vicinity of the pipeline route was set out in the annex, which was not available to the author.
2. It is stated several times that the pipeline passes through areas designated as residential in planning documents, or that the pipeline is approaching a residential area along a particular section. There is no numerical indication of how far away the buildings are or where they are planned to be located.

## Discussion

The “Discussion” section is entirely devoted to answering the research question concerning the required improvements to the consideration of MADs in the EIA process for the NG pipeline projects. First, the focus is on the MADs’ risk assessment approach, then attention is put on the recognition of the importance of public participation and consideration of social factors in pipeline projects, in the context of mitigating social conflicts fueled by local public concerns about the hazards posed by MADs. The discussion covers two levels: the correctness of transposing the 2014 EIA Directive to the Polish 2008 EIA Act and the fulfillment of legal requirements in practice when preparing the EIARs in Poland.

### The Approach to Risk Assessment of MADs

Tomaszkiewicz and Syryczyński (2015) questioned the correctness of the Polish legislator’s transposition of the EIA 2014 Directive to the 2008 EIA Act regarding the risk assessment of MADs pointing out several differences between the current EU and Polish EIA legislation. Their main critical remarks can be summarized as follows:

1. The Polish legislator talks about natural and construction disasters, while the EU legislator does not limit the set of disasters. As a result, a disaster due to a terrorist attack does not fall within the scope of the EIA, as it is neither a natural nor a construction disaster.
2. The EU legislator deals with the significant effects of the project, while the Polish legislator omits the adjective “significant”.
3. The EU legislator is concerned with the vulnerability of a project to the risk of MADs relevant to the given project, while the Polish legislator talks about identifying, analyzing and evaluating the risk of major accidents and natural and construction disasters.

Undoubtedly, the indicated differences in the wording of the EU and Polish EIA regulations may raise doubts about the correctness of incorporating the 2014 EIA Directive into Polish law. However, it should be emphasized that pursuant to Article 288 of the EU Treaty on the functioning (2012), MSs have a choice of the form and methods of implementing directives into national law. The provisions of the directive bind them as to the effect to be achieved and the deadline by which the transposition is required to take place. The question, therefore, arises as to whether the Polish legislator’s transposition allows the objective of the directive to be achieved. An in-depth interpretation of the provisions, learning about their context, place in the system, functions and objectives they serve leads to the following observations:

1. The limitation by the Polish legislator of the set of man-made disasters to construction disasters makes the difference between the national and EU regulations significant in some cases, e.g., in the context of the risk of terrorist attacks. The use of a different term defined in the Polish legal system, “technical disaster”, instead of “construction disaster”, which, according to Article 3(3)2 of the Act 2002/62/558 (2002), can also be caused by cyber events and acts of a terrorist nature and in practice includes construction disasters, seems a better solution, especially since the term “construction disaster” was introduced in opposition to the term “natural disaster” in the same legislation act (Table 5). It is worth adding that Mahon (2018), pointing out a similar problem, i.e., the lack of legal definition of the term of man-made disasters in the EU and UK EIA regulations, stated that British EIA practitioners move forward with the inclusion of MADs in their EIAs typically assessing a project based on an elaborate list of accidents and disasters, including low-probability ones such as a list issued by the Cabinet Office (2015) or a list available at the Red Cross website.

**Table 5:** Definitions of the Terms “Construction Disaster” and “Technical Disaster” in the Polish Legislation

Regulation	Definition
Act 1994/89/414 1994: Article 73	A construction disaster is “an unintentional, violent destruction of a construction building or a part of a building, as well as structural elements of scaffolding, elements of forming devices, sheet piling, and excavation lining”.
Act 2002/62/558 2002: Article 3(1)3	A technical disaster is “a sudden, unforeseen breakdown or destruction of a structure, technical equipment, or system of technical facilities, resulting in an interruption of their operation or a loss of their characteristics”.
2. Article 59 of both versions of the EIA Act (i.e., the 2008 EIA Act and 2015 EIA Act) states that “an EIA is required for [two types of] planned projects likely to have a significant impact on the environment”. If it is noted that the Polish legislator, when defining these projects, speaks of “significant impact”, this means that the EIA procedure should also focus on the significant environmental effects of a project and not on all effects. If so, there is no conflict between national Polish law and the standards of the EU EIA Directive.
3. The practice of conducting EIAs for specific projects in the UK (EIAR 2019d) and Ireland (EIAR 2020a, 2023), where the provisions of the EIA Directive concerning the MADs have been literally transposed to the national legislation (in UK the transposition took place before the Brexit), shows that the methodology for assessing the vulnerability of a proposed project to MADs risks uses a risk-based approach, which covers three stages: risk identification, risk analysis and risk evaluation. Taking this into account, the practical effect of the 2014 EIA Directive’s standard in Polish national law is assured if the legal requirement of risk identification, risk analysis and risk evaluation is fulfilled. It should be noted, however, that doubts could be avoided by using strict transposition (word for word).

Regarding the fulfilling the EIA legal requirements concerning the risk assessment of MADs, the study showed that Polish EIA practitioners are aware of the new EIA regulatory framework and that EIARs must include new content. However, even three out of five EIARs prepared after the entry into force of the 2015 EIA Act, i.e., after 1 of January 2017 (which discussed the issue of the probability of MADs and their consequences for people and the environment in detail), neither introduced the concept of risk, understood as the combination of the probability of hazards//impacts and their consequences//effects, nor presented the results of the analysis of such defined risks carried out as part of the EIA process or at earlier stages of the project location.

Although, in principle, the results of this study are exploratory and limited in their generalizability (due to the way EIAR was selected, i.e., based on their availability and sample size, i.e., the set consists of eight documents), the results obtained provide important guidelines on existing gaps and possible needs in EIA practice. There are at least two arguments that ease concerns about representativeness. Firstly, Pasikowski (2015) emphasizes that the issue of sample size in qualitative research, as opposed to quantitative research, is a secondary matter. Secondly, the results obtained in the present study are in line with the results of the author’s previous research, in which a set of 20 selected spatial management plans for NG pipeline routes and SEA reports concerning the assessment of the risks to both people and the environment resulting from the NG pipeline accidents was analyzed (i.e., it was found that although the issue was addressed in the SEA reports to a limited extent, the risk, defined as the probability of hazards in combination with their consequences, was not considered in the SEA process (Markiewicz 2023)). It also needs to be stressed that EIARs for NG pipeline projects are rarely published online. Gathering a larger number of documents and analyzing them would require launching a project involving several people from various institutions and obtaining financial support, which was not possible at the time this study was initiated.

When looking for an explanation for the lack of assessment of the risk of MADs, defined as the probability of hazards//impacts in combination with their consequences//effects in the EIARs, two reasons come to mind. The first is the lack of definition of risk in the Polish EIA legislation. The second is the lack of general guidelines for integrating MADs risk assessment within the EIA process as well as the stand-alone sector-specific guidelines for assessing the risk of MADs on pipelines transporting hazardous materials (including NG pipelines), which could be used to support LUP decisions in addition to the arbitrary DMINs that are used in Poland today. The lack of the definition of risk in the Polish EIA legislation does not pose a problem as its definition is in Article(32c) of the Act 2001/62/627 (2001) and according to the linguistic interpretation of the law, in the absence of a legal definition in the EIA regulations, it should be assumed that the legislator used the term in the sense defined by the source definition. To address other shortcomings, it would be beneficial to develop the guidelines documents at the outset.

In terms of general guidelines on the integration of MADs risk assessment in the EIA process, all approaches described in the “Approaches for the risk assessment of MADs within the EIA” section (and probably others not included here) could potentially be useful. Still, in the author’s opinion, it seems appropriate to consider applying an approach dedicated to the EIA, i.e., the methodology from the IEMA primer (2020). The main advantages of this methodology are: structured assessment approach, efficiency and proportionality in assessment, making efficient use of available risk assessment method, providing definitions of key terminology. Disadvantages include a lack of an inventory of accidents and disasters (with the result that application of the methodology may be difficult in countries where such an inventory does not exist), and few examples of application in the UK. The methodology, despite a certain level of generality, contains integration with the current UK legislation (for example classification of risk according to the ALARP principle, significance criteria for MADs which are based on criteria for notification of major accident hazards from Annex VI of the Seveso III Directive transposed to the UK regulations, UK applicable regulations surrounding developments). When applied in the UK, this is an advantage; however, when applied in another country, it should be adapted to the country’s national realities and regulations. Applying the methodology in Poland would require the following: presenting the Polish useful legislative background (i.e., the regulations surrounding developments),introducing risk acceptability criteria and a risk classification suitable for Polish conditions, and the gradual building of the sample of Polish case studies that demonstrate the methodology application. When it comes to further development of the IEMA methodology, it would be worthwhile to introduce a risk classification using a “Probability//Consequence Matrix” technique, the application of which, as the example of the Irish EIA carried out for the DART+Westilway extension project shows (EIAR 2020a), makes the results transparent.

Regarding the stand-alone, sector-specific guidelines for assessing the risk of MADs on pipelines transporting hazardous materials to support the LUP decisions, it is suggested to consider the GESIP methodology (GESIP 2014) used in France (Markiewicz 2023). However, before making decision, it is important to carefully analyze how this method fits into Polish legal system. Application of the GESIP methodology for the safety study for an NG pipeline (DN 500 mm, MOP 2.5 MPa) localized in Paris suburbs is given in Chatelet (2019). As modelling the environmental impacts of gas releases to the atmosphere is challenging and it is treated in a simplistic manner in the GESIP methodology, it is worth developing this element in the future.

### The Relevance of Public Participation and Consideration of Social Factors in EIA for NG Pipeline Projects, with a Focus on Mitigating Social Conflicts, Particularly those Fueled by Local Community Concerns about the Hazards Posed by MADs

In statutory recognition of the importance of public participation and consideration of social dimension of the project’s impacts, Polish legislation it is not only in line with EU law but goes a step ahead by requiring the EIARs to contain the analysis of the possible social conflicts (which has been present in the Polish EIA regulations since 2000). Noticing this added value is important because there is an ongoing discussion among some EIA practitioners (Mezzalama et al. 2014; Paterson 2025) and researchers (Dendena and Corsi 2015; Larsen et al. 2015; 2018) on whether EU legislation is sufficient to introduce a social dimension into project’s impacts. Although the prevailing view is that the UE EIA legislation has the potential to deal with social impacts, provided practice improves (Dendena and Corsi 2015; Larsen et al. 2015; 2018) the issue of lagging legislative requirements is also brought up (Mezzalama et al. 2014; Paterson 2025).

Regarding public participation and consideration of the social factors in the EIA practice for NG pipeline projects, with a focus on mitigating the social conflicts, particularly those fueled by the local community concerns about the hazards posed by MADs, the study confirmed that both Polish EIA practitioners and the proponent, the Gas System company, recognize the importance of both activities. However, there is room for improvement in this regard, which is directly related to the recommendations concerning the assessment of the risk of MADs presented in the previous section. Assessing risk, defined as the probability of hazards in combination with their consequences, and checking whether risk values are within acceptable limits, in addition to the arbitrary, minimum distances from the NG pipeline to buildings and other structures that are used in Poland, would not only strengthen the confidence in the assessment process but also can contribute to the mitigation of social conflicts. This is particularly concerning for sections where the NG pipeline approaches or crosses residential areas.

## Conclusion

In brief, the research results show that the issue of MADs are considered in all analyzed Polish EIARs for NG pipeline projects. Polish EIA practitioners are aware of the new EIA regulatory framework concerning risk assessment of MADs, which came into effect on January 1, 2017, and the analyzed reports prepared after this date generally contain more detailed information on MADs. None of the reports introduced the concept of risk, understood as the probability of occurrence of hazards//impacts in combination with their consequences//effects, nor did they present the results of the analysis of such defined risk carried out as part of the EIA process or at earlier stages of project location. In the author’s opinion, in addition to the arbitrary minimum distances from the gas pipeline to buildings and other structures used in Poland (DMIN), it would be valuable to consider such risk assessment results and check whether the risk values are within acceptable limits. Therefore, it is recommended that Poland develop separate, sector-specific guidelines for MAD risk assessment on pipelines to support LUP decisions, as well as general guidelines for incorporating MAD risk assessment into the EIA.

Although Polish EIA practitioners and proponents recognize the importance of public participation and consideration of social factors in the EIA practice, in the context of mitigating social conflicts fueled by concerns about hazards posed by MADs, there is still room for improvement in this regard. It relates directly to the recommendations concerning the presentation of the results of the risk assessment of MADs in the EIARs. It would strengthen confidence in the EIA and potentially contribute to mitigating conflicts.

In addition, it is suggested that the Polish EIA legislation be modified and a set of man-made disasters analyzed in the EIA be expanded to include consideration of “technical disasters” in place of “construction disasters”.

The research results presented in this article are considered to have useful implications for MADs control on pipelines transporting hazardous materials, LUP and EIA in Poland (by suggesting some improvements in legislation and practice) but also in some other countries in similar situation, in particular MSs (by providing a baseline for comparisons and encouragement for revision of their national EIA legislation and practice).

Regarding future research, two ideas are worth considering. Considering that the current study was limited to NG pipeline projects, it would be beneficial to investigate how MADs are considered in EIARs for other types of hazardous projects. As the current research was limited to considering only the social impacts related to the hazards posed by MADs on NG pipelines, it would be interesting to find out how the social impacts are generally considered in the EIARs for hazardous projects which usually are the most conflictual ones, and whether the social impacts important to the local community are properly addressed. There would be added value in organizing these two projects in an international setting, especially as the results could be used to inform the suspended discussion on the need for EU-level legislation on the control of MADs on pipelines transporting hazardous materials (in addition to the already existing Seveso III Directive, which regulations cover hazardous establishments) and the current debate on the extension of the provisions on social impact assessment in the currently binding 2014 EIA Directive.

## Data Availability

No datasets were generated or analysed during the current study.

## Appendix

Polish legal regulations regarding the consideration of MADs in the EIA in relation to the EU law.

A review of the Polish legal regulations, was focused on changes in Polish EIA legislation, i.e., the 2008 EIA Act, which resulted from the implementation of the 2014 EIA Directive. The new Polish EIA legislation, i.e., the 2015 EIA Act became effective on 1 of January 2017.

Within the EU, EIA legislation goes back almost forty years. The first so-called EIA Directive, issued in 1985, was modified in 1997, 2003 and 2009, consolidated in 2011 and modified one more time in 2014. MSs were required to implement the changes in legislation. The latest EIA Directive, which came into force on 15 of May 2014, has been in force since 16 of May 2017. MSs had four years to adopt their transposing legislation.

Regarding the safety of the hazardous materials transported by pipelines, this issue was already explicitly considered in the first edition of the EIA Directive (Directive 85/337/EEC 1985; hereafter referred to as the 1985 EIA Directive). The provisions concerning the issue have been further developed in its upgraded versions. Initially, the gas and oil pipelines were included in the catalogue of projects that were not likely to have a significant effect on the environment in every case (1985 EIA Directive: Annex II(10 h)), but in 1997 the pipelines with a diameter and length exceeding 800 mm and 40 km, respectively were regrouped and classified as projects with significant effects on the environment, which must therefore, in principle, be systematically assessed (Directive 97/11/EC 1997, hereafter referred to as the 1997 EIA Directive: Annex I(16)).

In 1997 the eligibility criteria for conducting an EIA was also introduced, which included, among others, the risk of accidents (1997 EIA Directive: Annex III(1 f)).

In 2014, amendments were made to the 1997 EIA Directive, of which the most important concerned extension of the requirements for the EIA process such as directly taking into account project’s vulnerability to MADs, determining the potential of these MADs to cause likely significant adverse effects to people and the environment as well as proposing measures to mitigate and prevent these effects and to describe the details of preparedness and propose response to such emergencies (2014 EIA Directive: Recital 15 and Article 3(2)). Also, the obligations for the submission of information for the EIAR were extended in order to comply with the above-mentioned provisions (2014 EIA Directive: Annex IV(5 d, 8). In addition, the requirements for qualifying for an EIA were amended, to take into account the risk of project-specific disasters (2014 EIA Directive: Annex III(1 f)).

Regarding the social dimension of project’s impacts, the EU EIA legislation requires to some extent to pay attention on them (i.e., the 1985 EIA Directive required in Article 3(1) to consider how the project influence, among others, on “human beings […], material assets, cultural heritage”, and now the 2014 EIA Directive keeps this requirement with the change of the wording of “human beings” to “human population and health”), but this requirement is not very explicit. It is worth to add that also Recital 9 of the 2014 EIA Directive acknowledges the economic and social relevance of good land management. Although EU EIA legislation contains extensive provisions that ensure the opportunity for public participation in environmental decision-making from the beginning of the EIA procedure at each of its stages before a decision is taken (these were already present in the 1985 EIA Directive (Articles 6- 9) and have been progressively extended with new arrangements for informing and consulting the public in subsequent editions of the EIA Directives), none of them directly address the issue of social conflicts.

It is worth notice that there are no specific EU legislation on LUP measures that enhance the safety of the NG pipeline itself and the safety of the commune in the vicinity of this installation. The requirement of keeping the safe distance between the hazardous sites and the residential and other sensitive areas in the Article 13 of the Seveso III Directive do not cover the pipelines outside the so called Seveso establishments. Although there have been discussions in the past on the need for EU-level legislation on the control of major accidents on NG pipelines (COWI report 2011; Papadakis 2000), this idea has not been realized.

In Poland, the classification of projects for the EIA process is identical to that in the EU, established by the 1997 EIA Directive. In the case of oil and gas pipelines, the same pipeline parameter limits (i.e., pipeline diameter of 800 mm and its length of 40 km) apply to distinguish the two types of projects. This categorization of gas transportation pipelines was introduced in Poland in 1998 by the Ordinance 1998/93/589. It has been maintained in subsequent Ordinances concerning this issue, i.e., Ordinance 2004/257/2573 (2004), Ordinance 2005/92/769 (2005), Ordinance 2007/158/1105 (2007), Ordinance 2010/213/1397 (2010b), Ordinance 2013/817 (2013b), Ordinance 2019/1839 (2019), Ordinance 2023/1724 (2023).

Regarding the project eligibility criteria for an EIA with direct reference to MADs, the 2008 EIA Act required consideration of the risk of major accidents (Article 61(1)(1e)), while the 2015 EIA Act has extended this provision to include also project-relevant natural and construction disasters (implementation of 2014 EIA Directive: Annex III(1 f)). As for the consideration of MADs in the EIA process of the particular project, the 2008 EIA Act did not directly refer to this issue, but required this process to identify, analyze and evaluate the direct and indirect effects of the project on the environment and population, interaction between these elements and possibilities and ways to prevent and reduce the negative impact of the project on the environment as well as the required scope of monitoring (Article 62(1-3)). It was only the 2015 EIA Act that directly referenced MADs requiring in Article 62 (1a) to identify, analyze and evaluate the risk of major accidents and natural and construction disasters (implementation of 2014 EIA Directive: Recital 15 and Article 3(2)).

As the information contained in the EIAR is concerned, the 2008 EIA Act required the document to include three elements directly related to the MADs, i.e., a description of the expected environmental impact of the options examined, including in the case of a major industrial accident, a characterization of the measures for avoiding, preventing, reducing or compensating adverse impact on the environment and a proposal of monitoring of the planned project’s impact (Article 66(6, 9, 16)). The 2015 EIA Act has widened the provision of Article 66 by adding Paragraph 1 g, which requires to include in the EIAR a description of the risk of major accidents or natural and construction disasters […], and expanded Article 66(6) to include natural and construction disasters (implementation of 2014 EIA Directive: Annex IV(5 d, 8)). It is worth explaining that according to the COWI report (2011) Poland is the only EU country where the definition of major accident created for the purpose of the Seveso III Directive, provided in Article 3(23) of the Act 2001/62/627 (2001), also covers transporting hazardous materials by pipelines outside a Seveso establishment by rail, road, watercourses, and pipelines. So, the adjective “industrial” is important here and was added due to the difference in defining a major accident in EU and Polish legislation.

Regarding the social dimension of the project’s impact, it is somewhat better handled in the Polish legislation in comparison to EU law. While the 2008 EIA Act listed “human health and living conditions, […] material assets, cultural heritage” (Article 62(1)) among the factors in relation to which the effects of a project must be assessed, the currently binding 2015 EIA Act, changed the wording to “population, including human health and living conditions[…], material assets, cultural heritage” (Article 62(1)). The added value of the Polish legislation is the assessment of project’s impacts on “living conditions”. What is more, in addition to the specific provisions on public participation in environmental protection, including in the EIA process, contained in Part III of both Polish EIA Acts (in particular, Article 29 stipulates that everyone has the right to submit comments and proposals, Articles 33-38 set out the conditions for public participation, from which obligations of the authority conducting the proceedings to inform and consult the public arise, and Article 33a added to the 2015 EIA Act clarifies the manner of making the above information public) Article 66 (1)(15) requires that the EIA Report contains an analysis of possible social conflicts related to the project.

In Poland pursuant to Article 61(2), the EIA of a project, which forms part of the procedure for issuing a decision on environmental conditions (DEC), is carried out by the authority competent to issue that decision. As the projects in the sector of liquefied NG regasifiction terminal is concerned, the competent authority for issuing the DEC is the Regional Director of Environmental Protection (RDEP) (Article 75(1)(1 f)) and if the project extends beyond the area of a single voivodship, the DEC shall be issued by the RDEP in whose area of jurisdiction the largest part of the area on which the project is to be implemented is located, after obtaining the opinion of the RDEP competent for the remaining area on which the project is to be implemented (Article 75(5)).

As for the LUP measures that enhance the safety of the NG pipeline itself and the safety of the commune in the vicinity of this installation in Poland, these include minimum distances from the NG pipeline to buildings and other structures (DMINs), hazardous zones related to the possibility of an explosive atmosphere in the workplace, and consultation zones along the pipeline route.

The values of DMINs for the pipelines built after 12 of December 2001, which are of interest in this study (hereafter called new pipelines), depend on the characteristics of the pipeline (including the construction material and the nominal pressure in the pipeline) and are scaled based on the of the control zone width and the location class of the pipeline (Ordinance 2001/97/1055 2001a; Ordinance 2013/640 2013a).

A control zone is a strip of land on each side of the pipeline, the center line of which is in line with the pipeline’s center line in which NG company takes measures to ensure pipeline durability and its proper operation. The concept was introduced in 2001 by Ordinance 2001/97/1055 (2001a). Initially, the term concerned only the new pipelines, but in 2013, it was extended to cover also the pipelines built prior to 12 of December 2001 (hereafter referred to as the old pipelines) (Ordinance 2013/640 2013a). The values of control zones for the new pipelines depending on the pipeline’s diameter and its nominal operating pressure ranges from 1 m to 12 m. It is worth mentioning that they are much smaller than the values of control zones for the old pipelines, which range from 50 m to 200 m. Limitations and bans on land usage in the control zones concern constructing the structures, planting the trees and performing any work (Ordinance 2013/640 2013a: Article 10(1, 2, 3)).

Three location classes have been distinguished, with class I representing the areas with the most intensive urbanization, class II- urbanized areas and class III - undeveloped land and areas with isolated single-family or farm buildings (Ordinance 2013/640 2013a: Article 7(2, 3, 4)).

For steel pipelines with a nominal pressure higher than 0.5 MPa for location classes I, II and III, the DMIN equals 50%, 100% and 150% of the width of the control zone, respectively. For all other pipelines, i.e., polyethylene pipelines with a nominal pressure of less than 1 MPa and steel pipelines with a nominal pressure of no more than 0.5 MPa for all three location classes, the DMIN equals 50% of the width of the pipeline’s control zone.

For the pipelines included in the study, i.e., for the new steel pipelines having a diameter equal to or greater than 700 mm and a nominal pressure greater than 8.4 MPa, the pipelines control zone is 12 m so DMINs for the location classes I, II, and III are 6 m,12 m and 18 m, respectively.

The risk assessment for gases distinguishes between three classes of explosive atmospheres in the workplace: hazardous zone 0 is a space where explosive atmospheres occur continuously, with high frequency or over a long period; hazardous zone 1 is a space where explosive atmospheres are likely to occur during regular operation of the installation; hazardous zone 2 defines a space where explosive atmospheres are unlikely to occur during normal operation of the installation or, if they do occur, for a short period (Ordinance 2010/138/931 2010a). Zabrzeski et al. (2017) reported that the IGG (Izba Gospodarcza Gazownictwa) (2015), IGEM (Institute of Gas Engineers and Managers) (2013) and PKN (Polski Komitet Normalizacyjny) (2016) standards are most used for the design of explosion hazard zones in Poland.

The provision for the consultation zone was introduced by the Act 2021/922 (2021) to the Act 2003/80/717 (2003) and started to bind on 27 of May 2021. The consultation zone is measured from the pipeline axis and extends perpendicular to the pipeline axis for a certain distance in each direction. Depending on the type of NG pipeline system (distribution or transmission network) and the pipeline diameter it can range from 25 m to 65 m. Consultations between stakeholders should address the land usage in this zone when developing a local spatial management plan or a draft of the decision on the conditions of development, where the area covered by these documents overlaps with the consultation zone. As explained, this provision directly concerns the LUP aspect rather than the EIA process itself, but it is presented here as they are related.

It should be noted that there are no stand-alone, sector-specific guidelines for assessing the risk of MADs on pipelines transporting hazardous materials to support the LUP in Poland.

## Material Availability

Materials analyzed in the study are included in the references cited.
